# Imaging the NADH:NAD^+^ Homeostasis for Understanding the Metabolic Response of Mycobacterium to Physiologically Relevant Stresses

**DOI:** 10.3389/fcimb.2016.00145

**Published:** 2016-11-08

**Authors:** Shabir A. Bhat, Iram K. Iqbal, Ashwani Kumar

**Affiliations:** Council of Scientific and Industrial Research - Institute of Microbial TechnologyChandigarh, India

**Keywords:** NADH:NAD^+^ homeostasis, Peredox-mCherry, metabolic state, *Mycobacterium tuberculosis*, tuberculosis pathogenesis, immunological stimulation, autophagy

## Abstract

The NADH:NAD^+^ ratio is the primary indicator of the metabolic state of bacteria. NAD(H) homeostasis is critical for *Mycobacterium tuberculosis* (Mtb) survival and is thus considered an important drug target, but the spatio-temporal measurements of NAD(H) remain a challenge. Genetically encoded fluorescent biosensors of the NADH:NAD^+^ ratios were recently described, paving the way for investigations of the metabolic state of pathogens during infection. Here we have adapted the genetically encoded biosensor Peredox for measurement of the metabolic state of Mtb *in vitro* and during infection of macrophage cells. Using Peredox, here we show that inhibition of the electron transport chain, disruption of the membrane potential and proton gradient, exposure to reactive oxygen species and treatment with antimycobacterial drugs led to the accumulation of NADH in mycobacterial cells. We have further demonstrated that Mtb residing in macrophages displays higher NADH:NAD^+^ ratios, that may indicate a metabolic stress faced by the intracellular Mtb. We also demonstrate that the Mtb residing in macrophages display a metabolic heterogeneity, which may perhaps explain the tolerance displayed by intracellular Mtb. Next we studied the effect of immunological modulation by interferon gamma on metabolism of intracellular Mtb, since macrophage activation is known to restrict mycobacterial growth. We observed that activation of resting macrophages with interferon-gamma results in higher NADH:NAD^+^ levels in resident Mtb cells. We have further demonstrated that exposure of Isoniazid, Bedaquiline, Rifampicin, and O-floxacin results in higher NADH:NAD^+^ ratios in the Mtb residing in macrophages. However, intracellular Mtb displays lower NADH:NAD^+^ ratio upon exposure to clofazimine. In summary, we have generated reporter strains capable of measuring the metabolic state of Mtb cells *in vitro* and *in vivo* with spatio-temporal resolution. We believe that this tool will facilitate further studies on mycobacterial physiology and will create new avenues of research for anti-tuberculosis drug discovery.

## Introduction

A highly flexible and robust metabolic system ensures survival of *Mycobacterium tuberculosis* (Mtb) inside the host (Boshoff and Barry, 2005; Lee et al., 2013; Gouzy et al., 2014). Mtb employs alternative and parallel metabolic pathways for *in vivo* survival, wherein it faces stresses such as limiting carbon sources, hypoxia, variable pH, reactive oxygen species, reactive nitrogen species, etc. (Pieters and Gatfield, 2002; Boshoff and Barry, 2005; Gleeson et al., 2016). Changes in the bacterial surroundings and the intracellular environment are continually sensed by Mtb to modulate its metabolism and to facilitate the transition between non-replicating persistence and active replication (Kumar et al., 2011; Bhat et al., 2012; Trivedi et al., 2012). Cellular levels of NADH and ATP are central determinants of bacterial energy state, and hence dictate the bacterial decision to enter persistence or active replication (Boshoff and Barry, 2005). An understanding of the sensory systems at molecular level and the plethora of metabolic pathways employed by Mtb is fundamental to the development of better diagnostic tools, effective vaccines, and potent drugs.

NAD(H) homeostasis is critical for the survival of pathogens and represents a potential drug target in many bacteria, including Mtb (Boshoff et al., 2008; Sorci et al., 2009; Rodionova et al., 2014). Estimates suggest that ~17% of the enzymatic reactions in Mtb use NAD(P)H as a cofactor (Beste et al., 2007). In Mycobacteria, the NAD(H) could be synthesized *de novo* from aspartate or scavenged through the salvage pathway. This *de novo* biosynthesis is essential in the survival of Mtb, but the bacilli could grow in the presence of exogenous NAD (Boshoff et al., 2008; Vilchèze et al., 2010) suggesting an important role of the salvage pathway in Mtb physiology. The salvage pathway is upregulated during hypoxia and in the lungs of mice infected with Mtb, where it plays a crucial role in Mtb survival (Boshoff et al., 2008; Vilchèze et al., 2010). The NADH:NAD^+^ redox pair is used for harvesting electrons from reduced carbon sources and feeds electrons into the electron transport chain through NADH dehydrogenase to generate a proton gradient. In Mtb, two types of NADH dehydrogenases are present: the proton pumping type (NDH-1) and the non-proton pumping type (NDH-2). NDH-1 is a multi-protein complex encoded by the *nuoABCDEFGHIJKLMN* operon, whereas NDH-2 is a single protein that exists in two isoforms encoded by *ndh* and *ndhA* (Weinstein et al., 2005). Interestingly, NDH-1 could be deleted from Mtb without functional consequences (Sassetti et al., 2003) and is downregulated during hypoxia and in the lungs of mice (Shi et al., 2005). NDH-2 is essential, and its inhibition by phenothiazine analogs can reduce mycobacterial growth *in vitro* and in mouse models and is therefore considered a drug target (Weinstein et al., 2005). During hypoxia, increased levels of NADH and reduced components of the electron transport chain are observed (Rao et al., 2008), which could be sensed by the redox sensor DosS (Kumar et al., 2007) to upregulate the Dos/dormancy regulon in Mtb (Honaker et al., 2010). Moreover, the NADH:NAD^+^ ratio is detected by the sensor protein Rex to regulate metabolism in Streptomyces (Brekasis and Paget, 2003). Rex is a transcription factor that regulates several genes involved in respiration including the cydABCD and rex-hemACD operons (Brekasis and Paget, 2003). Although a Rex homolog has not yet been described in Mtb, but Mtb uses a response regulator RegX3 that regulates expression of cydABCD (Roberts et al., 2011) and is responsive to oxygen levels (Singh and Kumar, 2015). Furthermore, accumulation of NADH leads to the induction of PknG, which activates the redox homeostatic system of Mycobacterium to regulate biofilm formation and augment survival of Mtb in macrophages (Wolff et al., 2015). NADH is also known to covalently bind to isoniazid to produce isonicotinic-NADH, which exhibits potent antimycobacterial activity (Rozwarski et al., 1998). Moreover, mutations in *ndh* lead to an accumulation of NADH levels that result in co-resistance to isoniazid and its analog ethambutol (Vilchèze et al., 2005). The above cited literature suggests a critical role of NADH in the physiology of Mtb.

Although NADH:NAD^+^ levels are considered to be the key indicators of cellular metabolism, appropriate tools to examine the spatiotemporal properties of NADH:NAD^+^ levels in bacteria, including Mtb, are lacking. The current methods of accessing bacterial NADH:NAD^+^ levels utilize cell lysis and presume that collective measurement in a bacterial population represents the metabolic state of individual cells and thus details of the heterogeneity of metabolic flux in a population have been largely ignored. These methods are further riddled with issues of low sensitivity and are unable to differentiate between free cellular NADH and protein bound NADH (Williamson et al., 1967; Huang et al., 2002; Schneckenburger et al., 2004; Zhao et al., 2011; Sun et al., 2012). Recently, genetically encoded fluorescent probes have been developed to measure the NADH:NAD^+^ ratio, enabling observation of the metabolic state in live cells with spatiotemporal resolution (Hung et al., 2011; Zhao et al., 2011; Bilan et al., 2014). One such NADH-NAD^+^ sensor is Peredox, in which the circularly permuted GFP T-Sapphire has been integrated with the NADH:NAD^+^ sensor Rex protein domains from *Thermus aquaticus* (Hung et al., 2011). Rex protein is a NADH:NAD^+^ ratio responsive transcription factor involved in repression of respiratory genes (Brekasis and Paget, 2003). This repression is regulated by NADH:NAD^+^ redox poise of cell and is dictated by the higher affinity of Rex toward NADH. Upon binding of NADH during high NADH:NAD^+^ conditions, the Rex protein undergoes conformational changes releasing the DNA and thus derepression of the respiratory flux. Several mutations were introduced in the Rex protein to minimize the DNA and NAD^+^ binding. These variants of Rex were fused with cpT-Saphire that produce significantly higher fluorescence upon binding to NADH (Hung et al., 2011). This fluorescence corresponds to NADH:NAD^+^ inside the cells. In order to normalize the sensor response, the Peredox sensor was fused to mCherry protein that does not change the fluorescence upon binding to NADH.This protein engineering has resulted in an efficient biosensor that does not require cell disruption and can measure metabolically relevant free NADH levels inside the mammalian cells. However, such a sensor has not yet been employed for measurement of NADH:NAD^+^ levels in bacterial cells.

In this study, the Peredox-mcherry sensor (Hung et al., 2011) was utilized for determining the NADH:NAD^+^ ratio in slow- and fast-growing mycobacteria. Changes in NADH:NAD^+^ were analyzed to assess the effect of antibiotics and other physiologically relevant stressors on mycobacterial metabolism. We have further employed this probe to analyze the NADH:NAD^+^ ratio in Mtb during infection in macrophages. These sensors could pave the way for acquiring new insights into the mechanisms of action of drugs and will aid in the development of new methods for discovering antimicrobials.

## Materials and methods

### Chemicals and drugs

All the inhibitors, chemicals and drugs were purchased from Sigma-Aldrich or as otherwise mentioned. Gibco® Dulbecco's Modified Eagle's Medium (DMEM) Glutamax™ medium and Fetal bovine serum (FBS) were obtained from life technologies. Bedaquiline was purchased from MedChem Express®. Middlebrook 7H9, 7H10, and 7H11 media and O-ADC were from BD biosciences. IFN-γ was procured from BD biosciences.

### Bacterial strains, media and cell culture conditions

RAW 264.7 (ATCC® TIB-71™) cells were obtained from ATCC and cultured in DMEM Glutamax medium supplemented with 10% heat inactivated FBS under 5% CO_2_ at 37°C. RAW 264.7 macrophages were activated with 400 units/ml IFN- γ for 2 h. After 2 h of treatment, the macrophage cells were infected with Mtb H37Rv (ATCC 27294) (transformed with pMV762-Peredox-mcherry) with MOI of 1:10 and incubated at 37°C for 3 h. The cells were washed thrice with warm 1X PBS to remove extracellular bacteria. *M. smegmatis* (Msmeg) strains Δ*mshA*, Δ*mshD*, Δ*mca* were kindly provided by William R. Jacobs (Albert Einstein College of Medicine, Bronx, NY, USA). While Msmeg strain Δ*sigH* was a kind gift from Robert N. Husson (Children's Hospital, Boston, MA, USA). Mycobacterial strains namely non-pathogenic mc^2^155 and pathogenic H37Rv were obtained from ATCC. Plasmid pMV762-Peredox-mcherry was introduced in these strains using the standard electroporation protocols (Goude et al., 2015) and the transformants were selected on 7H10 agar plates containing 10% of OADC and 50 μg/ml of Hygromycin B. These transformants were then picked and inoculated in 7H9 broth containing 10% O-ADC, Tween-80 (0.05–0.1%) and 50 μg/ml of Hygromycin B. The experiments were performed with the liquid cultures only. This episomal plasmid allows constitutive expression of the protein under *hsp60* promoter in Mycobacteria.

### Construction of plasmids

NADH:NAD^+^ measuring biosensor- Peredox-mCherry construct (Hung et al., 2011) was codon optimized for mycobacterial expression and cloned in *E. coli-Mycobacterial* shuttle vector pMV762 under the control of *hsp*60 promoter. PUC57-Peredox-mCherry was used as a template for the PCR amplification of Peredox-mCherry cassette using the forward primer: 5′-AATTGCCCATGGGATGGTCAAGGTCCCCGAAGCG-3′ and reverse primer: 5′- ATCGATAAGCTTTCACTTATACAGCTCATCCATACC-3′. The resulting amplicon was cloned in pMV762 vector between the NcoI and HindIII restriction sites. The plasmid sequence was confirmed by restriction digestion and partial sequencing.

### Treatments

Mycobacterial reporter strains (Msmeg/Mtb H37Rv expressing Peredox-mCherry sensor) were grown to mid-log phase (O.D_600_ = 0.5–0.8) in 7H9 medium containing 10% O-ADC, Tween-80 (0.05–0.1%) and 50 μg/ml of Hygromycin B and treated with different antibiotics, oxidoreductants or inhibitors. All the stock solutions were prepared in DMSO or otherwise mentioned. The growing bacterial culture, dispensed in 12-well plate and were exposed to antibiotics, oxidoreductants or inhibitors for 6 and 24 h and fixed with 4% paraformaldehyde, washed three times with PBS and mounted on glass slides using Slow Fade® to prevent from photobleaching. These slides were then subjected to confocal microscopy.

Antibiotics used in this study are isoniazid (0.2 ug/ml), rifampicin (0.2 ug/ml), pyrazinamide (2 ug/ml), ethambutol (5 μg/ml), levofloxacin (0.5 μg/ml), streptomycin (2 μg/ml), ofloxacin (1 μg/ml), bedaquiline (0.35 μg/ml) and clofazimine (0.2 μg/ml). Oxidoreductants with their working concentration are H_2_O_2_ (5 mM), cumene hydroperoxide (5 mM), paraquat (5 mM), Spermine NONOate (1 mM), DTT (2 mM), NaOCl (0.1%), hydroxyurea (10 mM), menadione (100 mM) and diamide (1 mM). Electron transport chain inhibitors used in this study were rotenone (140 μM) (as used in Zhang et al., 2003), oxaloacetate (2 mM), Antimycin-A (5 μM), KCN (0.1 μg/ml) and bedaquiline (0.35 μg/ml).

### Fluorimeter based measurements

Bacterial cultures harboring pMV762-peredox-mcherry were inoculated in 10 ml 7H9 medium containing 10% OADC, Tween-80 (0.05–0.1%) and 50 μg/ml of hygromycin and grown to mid log phase (O.D_600_ = 0.5–0.8). 200 μL of this growing culture was dispensed in each of the wells of a 96 well flat bottomed plate. These cultures were then independently exposed to antibiotics/inhibitor for 6/24 h and then the spectra were recorded in BioTek® hybrid fluorimeter using absorption wavelength of 400 and 587 nm, and emission was recorded at 510 and 615 nm, respectively (Hung et al., 2011). The ratio of fluorescence emissions at 510 to 615 nm (green/red) was plotted against antibiotics/inhibitors to estimate the levels of NADH/NAD^+^ ratio inside the bacterial cells.

### Infection in macrophages

For infection in macrophages, RAW 264.7 macrophage cells were seeded on cover slips, treated (activated or naive), infected with Mtb H37Rv Peredox reporter strain as per the standard protocol of Mtb infection. Briefly Mtb H37Rv reporter strain was grown to log phase and colony forming units were calculated. Glycerol stocks from these cultures were prepared in freezing media (15% glycerol in 7H9 media) and stored at −80°C until used. In order to infect macrophages, an aliquot of bacterial stock was thawed at room temperature. After breaking of bacterial clumps, bacterial suspension was made in antibiotic free DMEM media. This bacterial suspension was used to infect macrophages for 3 h (MOI = 10). Samples were fixed with 4% PFA for 10 min at room temperature. The samples were mounted with SlowFade® mounting reagent and analyzed using confocal laser scanning microscope (CLSM).

### CLSM imaging and ratiometric analysis

Mycobacteria grown *in vitro* in 7H9 medium containing 10% of OADC, Tween-80 (0.05–0.1%) and 50 μg/ml of Hygromycin B after treatment with different stressors and at different time points, the aliquots were taken from the culture and fixed with 4% paraformaldehyde (PFA) for 15 min and washed thrice with PBS and then resuspended in PBS. A drop of fixed culture was put on glass slide and a cover slip mounted over it. Similarly, the infected RAW 264.7 cells were seeded on cover slips and then fixed with 4% PFA. The fixed cells were washed three times with PBS and mounted on glass slides using SlowFade® as antifade reagent. These slides were then subjected to CLSM. Images were acquired using A1R Nikon equipped with 60X oil objective, through CCD camera connected to the computer running NIS elements software. The images were captured at the resolution of 1024 × 1024 pixels or 512 × 512 pixels with the pinhole of 1.0 by excitation with 405 nm laser collecting the emission using 525/50 nm filter, mCherry image was captured by exciting it with 561 nm laser and emission at 625/50 nm filter. The ratiometric image was processed by dividing the image captured at 405 nm with the image captured at 561 nm excitations (Green:Red ratio) depicting the NADH:NAD^+^ ratio inside the bacterial cells.

### Statistical analysis

Data shown are representative of the at least three biological experiments performed in technical triplicates. The data are presented as mean ± SEM and significance was determined either by applying *Student's t-test* or one way ANOVA followed by *Dunnett's* test to compare columns. *p*-value ≤ 0.05 was considered significant or otherwise mentioned.

## Results

### Development of a mycobacterial biosensor for determining the NADH-NAD^+^ redox state in live cells

**Figure 1:**
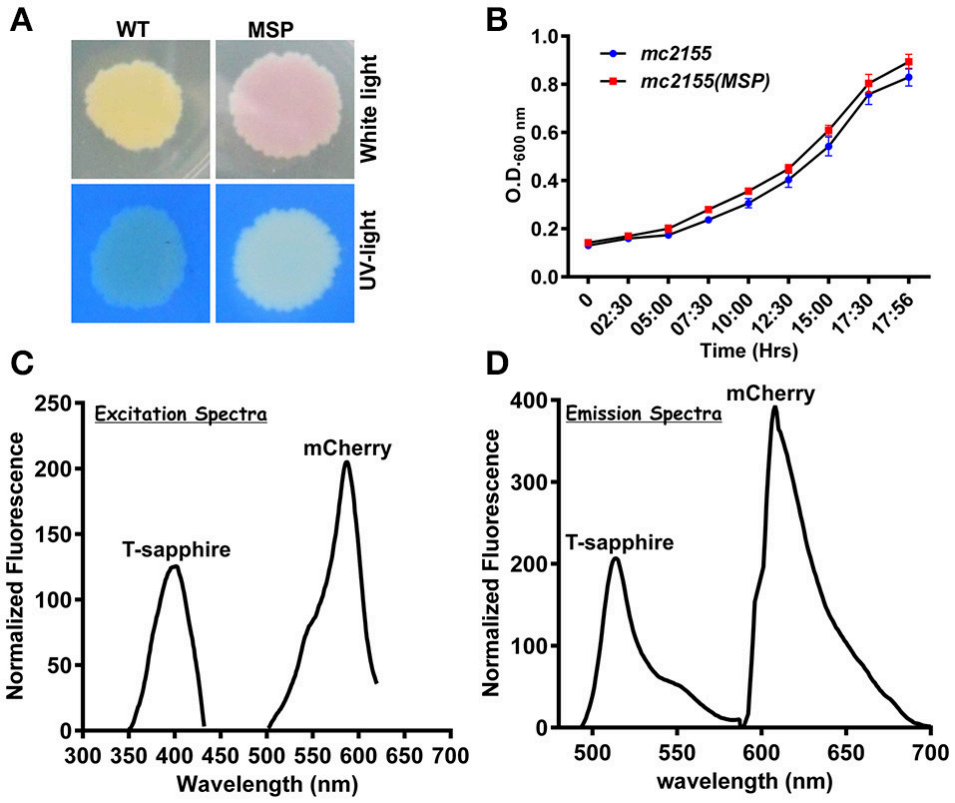
**NADH:NAD^**+**^ sensor (Peredox) was overexpressed in ***Msmeg*** mc^**2**^ 155 using IMT100 with no visible effects on bacterial growth. (A)** Msmeg expressing the reporter (MSP) colony on 7H10 agar plate under normal and UV-light demonstrating expression of the sensor. WT- Msmeg having pMV762; MSP-Msmeg containing IMT-100 and overexpressing Peredox. **(B)** Growth curve of Msmeg reporter strain (MSP) and Msmeg transformed with vector pMV762 only. **(C,D)** Representative excitation **(C)** and emission spectra **(D)** of Msmeg overexpressing Peredox-mCherry sensor showing excitation_max_ at 400 and 587 nm, respectively, and emission_max_ at 510 and 615 nm, respectively. T-sapphire indicates the fluorescence due to NADH:NAD^+^ responsive Peredox and mCherry indicates the fluorescence due to NADH:NAD^+^ unresponsive mCherry.

**Figure 2:**
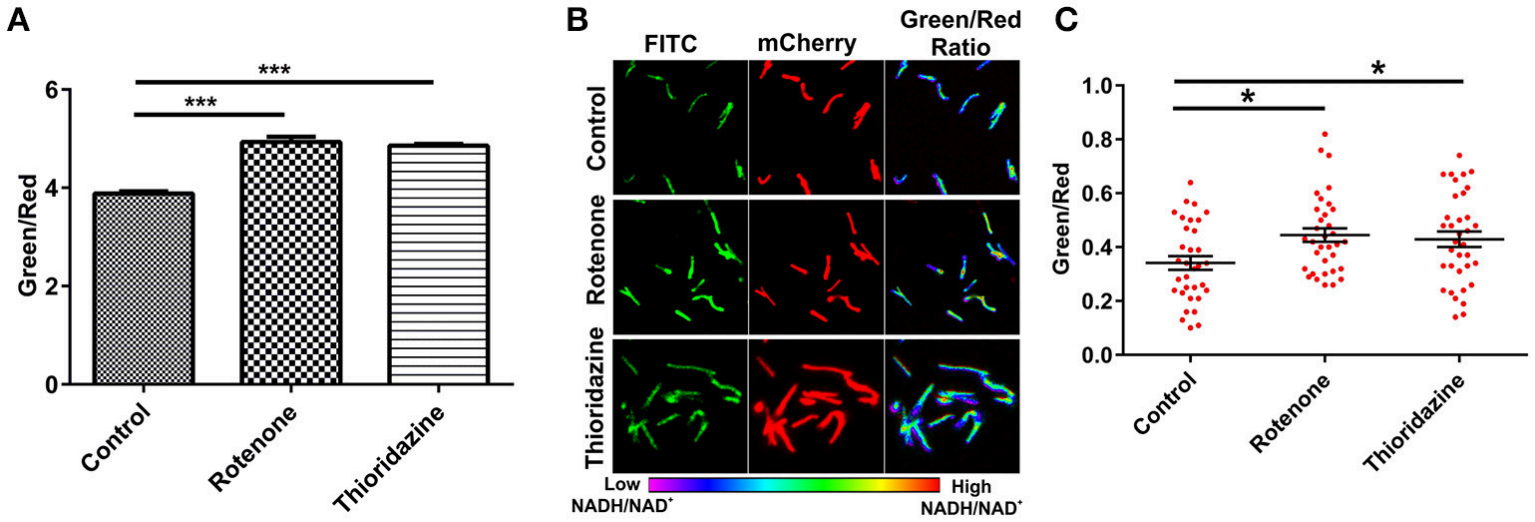
**Peredox expressing Msmeg strain responds to changes in the cytoplasmic NADH:NAD^**+**^ ratio**. Msmeg reporter strain was subjected to fluorometric **(A)** and confocal analysis **(B,C)** after treatment of log phase growing culture with different inhibitors (rotenone-140 μM and thioridazine hydrochloride- 40 μM) for 6 h; scatter plot of single cell ratios (red dots) after analysis of confocal images was plotted using GraphPad Prism® software. Images were captured using CLSM with excitation at 405 and 561 nm lasers, respectively. Captured images were used for the calculation of the ratio images. The green:red ratio was assigned a pseudocolor for presentation of the NADH:NAD^+^ levels in individual cells. The color scale for the ratio values indicates low and high NADH:NAD^+^ ratios. The data are presented as the mean ± SEM. Significance was determined by one-way ANOVA followed by *Dunnett's test*. ^*^p < 0.05, ^***^p < 0.001. **(A–C)** The data are representative of at least three independent biological experiments performed in triplicate.

**Figure 3:**
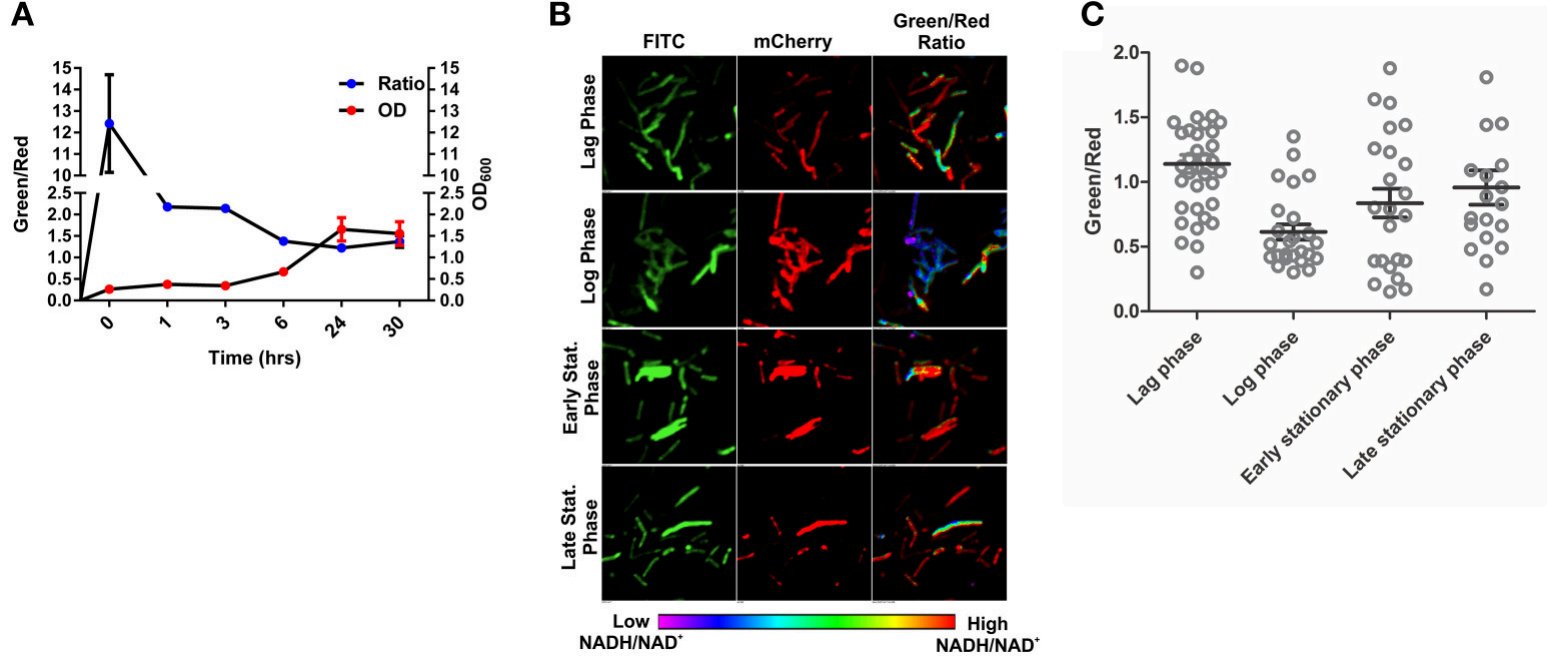
**Changes in NADH:NAD^**+**^ ratios during growth of mycobacteria. (A)** Msmeg reporter strain growth curve correlated with NADH:NAD^+^ ratios calculated using Fluorimeter. Green/red ratio on left Y-axis showing the NADH:NAD^+^ ratios and growth (OD_600_) is indicated on right with respect to time. **(B)** Representative confocal images (with their pseudo-colored images) of bacteria taken from different growth phases of the growth curve. **(C)** Scatter plot of bacteria from the different growth phases showing the heterogeneity in population.

To develop an accurate and non-invasive biosensor with spatiotemporal resolution of the NADH:NAD^+^ redox state in mycobacteria, we cloned and constitutively expressed codon optimized Peredox (Hung et al., 2011) in Msmeg using the mycobacterial shuttle vector pMV762 with an *hsp60* promoter (Steyn et al., 2003) (Figures S1A,B). The presence of Peredox in Msmeg resulted in pink colored colonies under visible light due to the presence of mCherry in the probe, and green colored colonies (due to T-Sapphire) upon exposure to UV light (Figure 1A). Importantly, we observed that the overexpression of Peredox in Msmeg does not affect its growth rate (Figure 1B). The expression of the probe in Msmeg was confirmed with florescence spectra at an excitation peak at 400 nm (Figure 1C) and an emission peak at 510 nm (Figure 1D). Peredox also contains an mCherry domain for normalization that results in another excitation peak at 585 nm and an emission peak at 615 nm. These spectral features are consistent with the reported spectra of Peredox (Hung et al., 2011). To determine if the Msmeg Peredox strain could detect changes in NADH:NAD^+^ levels in real time, we utilized an NDH-1 inhibitor (rotenone) and an NDH-2 inhibitor (thioridazine). The inhibition of these NADH dehydrogenases results in an increase in the intracellular NADH levels (Rao et al., 2008) that leads to an increase in the green (405/515) fluorescence of the probe (T-sapphire), while the mCherry fluorescence intensity (585/615) serves as a normalizing control (Hung et al., 2011). Thus, the increase in NADH:NAD^+^ levels results in an increase in the green:red ratio. Upon treatment with both rotenone and thioridazine individually, a significant increase in the green:red ratio was observed (Figure 2A), suggesting that both of the two NADH dehydrogenases instill electrons from NADH into the electron transport chain (ETC). These studies were repeated at the single cell level using confocal laser scanning microscopy (CLSM). With CLSM, the ratio of NADH:NAD^+^ is measured by independently quantifying green and red florescence and then computing the green:red ratio. These ratios are then assigned a pseudo-color for visualization in every single bacterium under the fields of observation. The results obtained in these experiments are in agreement with the data obtained from spectrofluorometry; however, these data shed light on the heterogeneity of the mycobacterial population in the culture (Figures 2B,C). Bacteria have been shown to display a large range of the NADH:NAD^+^ depending on the metabolic state. High NADH:NAD^+^ is observed in anaerobic conditions and low NADH:NAD^+^ is observed during aerobic growth (de Graef et al., 1999). To further study the dynamics of NADH:NAD^+^ during growth, we analyzed the metabolism of Msmeg during lag, log and stationary phase. We observed that during the lag phase, a high NADH:NAD^+^ ratio is maintained, but as the Msmeg cells start replicating, higher NAD^+^ levels are needed to maximize energy production for growing cells. NADH levels rise again in the stationary phase (Figure 3A). These studies were further confirmed at the single cell level using CLSM (Figures 3B,C).

### Effects of inhibition of the electron transport chain and the disruption of PMF on NADH:NAD^+^ levels in mycobacteria

**Figure 4:**
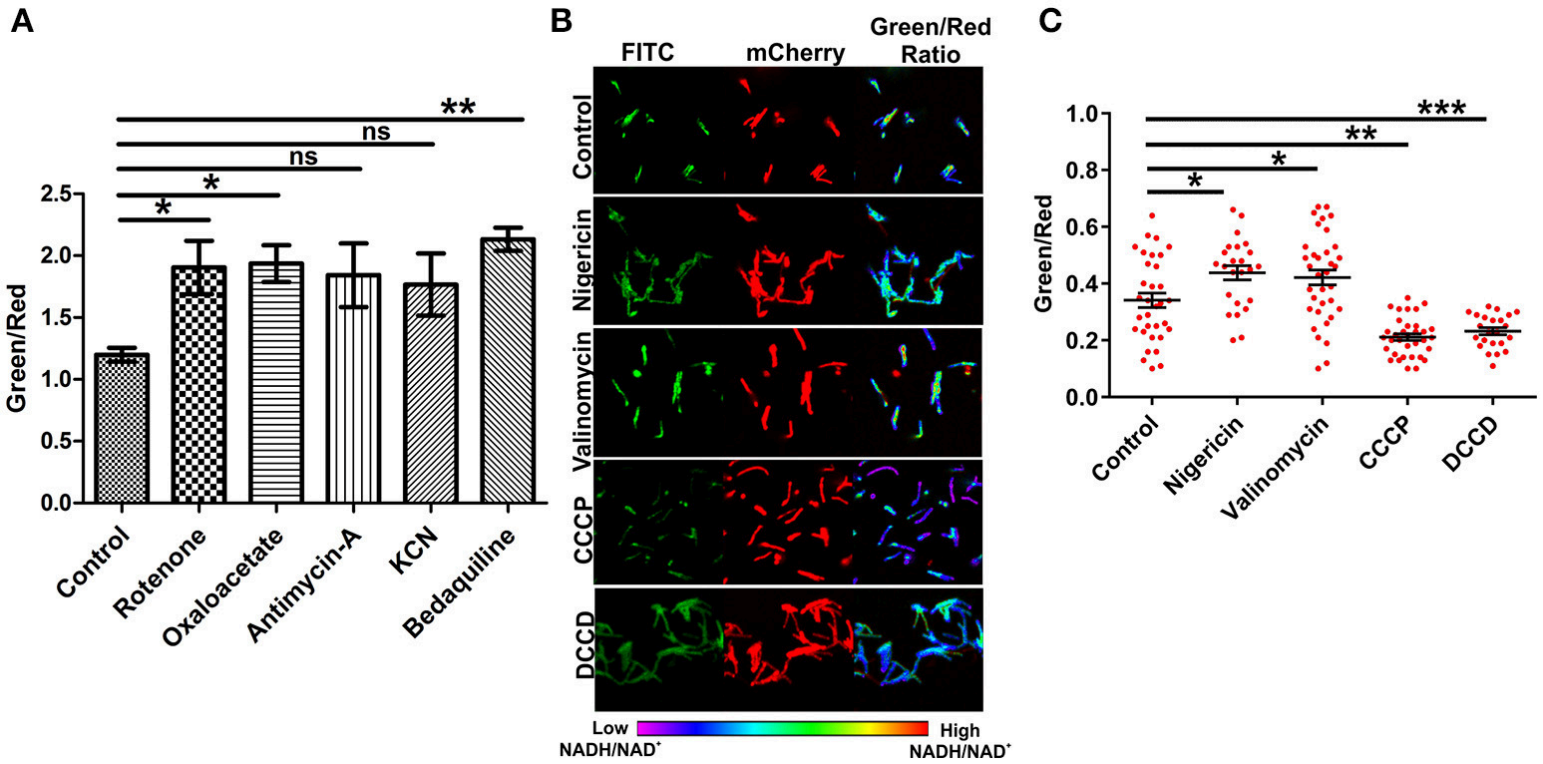
**Inhibition of the electron transport chain perturbs NADH:NAD^**+**^ ratios in mycobacterial cells. (A)** Fluorimetric data (mean ± SEM, *n* = 3) after 3 h of treatment of log phase cultures of Msmeg with different inhibitors of the electron transport chain namely complex-I (rotenone-140 μM), complex-II (oxaloacetate-2 mM), complex-III (antimycin-A-5 μM), complex-IV (KCN-0.1 μg/ml) and complex-V (bedaquiline-0.35 μg/ml), acquired using a Hybrid fluorimeter and data were plotted using GraphPad Prism® software. **(B)** The log phase cultures of Msmeg were treated with inhibitors of Δψ (nigericin-10 μM), ΔpH (valinomycin-5 μM), PMF (CCCP-10 μM and DCCD-100 μM) for 6 h and CSLM was performed. Confocal Images were captured in CLSM with excitation at 405 and 561 nm. Captured images were used for the calculation of ratio images. The green:red ratio was assigned a pseudocolor for the presentation of NADH:NAD^+^ levels in individual cells. The color scale indicates low and high NADH:NAD^+^ ratios. **(C)** Scatter plot of single cell ratios after analysis of confocal images. Data shown (mean ± SEM) are representative of at least three experiments performed independently in triplicate. Significance was determined by one-way ANOVA followed by *Dunnett's test*. ^*^p < 0.05, ^**^p < 0.01, ^***^p < 0.001 and ns, not significant.

The electron transport chain (ETC) plays a major role in the generation of a proton gradient in bacterial cells. NADH dehydrogenases are referred to as complex I of the ETC. Inhibition of the ETC at any stage should result in increased levels of NADH. In an effort to examine the effects of inhibiting ETC components on NADH levels, we exploited inhibitors of complex I, II, III, IV, and V. Interestingly, we observed a significant increase in the green/red (NADH:NAD^+^) ratio with the inhibition of complex I, II, and V. These findings are consistent with earlier observations that antimycobacterial agent bedaquiline (an inhibitor of complex V of ETC) leads to increased NADH levels in Mtb (Berney et al., 2014; Koul et al., 2014). We also consistently observed an increase in the ratio after inhibiting complex III and IV but this increase was not statistically significant (Figure 4A). Using spectrofluorometric and confocal microscopy approaches with the Msmeg reporter strain (Figures 2, 4A), we have been able to demonstrate that the blockade of ETC results in the accumulation of NADH levels in the cytoplasm of mycobacterial cells.

ETC is an integral component of the bacterial machinery involved in generation of the proton motive force. To determine the effect of disrupting the membrane potential (Δψ) through nigericin or the proton gradient (ΔpH) through valinomycin on NADH:NAD^+^ ratio, we treated the logarithmically growing Msmeg Peredox culture with these specific inhibitors for 6 h, and measured the NADH:NAD^+^ levels. Interestingly, we observed that the disruption of either the Δψ or the ΔpH resulted in increased levels of NADH:NAD^+^ (Figures 4B,C). However, upon disruption of both (collective known as proton motive force) the Δψ and ΔpH together (with CCCP or DCCD), a significant decrease in NADH:NAD^+^ was observed (Figures 4B,C). Such changes may have arisen due to oxidation of the NADH to NAD^+^ upon disruption of proton motive force by CCCP or DCCD. Since, confocal microscope was not available in the biosafety level 3 facility, we also analyzed if fixing the Msmeg cells using 4% paraformaldehyde (PFA) affect the measurement by Peredox reporter strains. We observed that fixing does not affect the measurement by Peredox reporter strain (Figure S2).

### Effect of oxidative and nitrosative stress on NADH:NAD^+^ in mycobacterium

**Figure 5:**
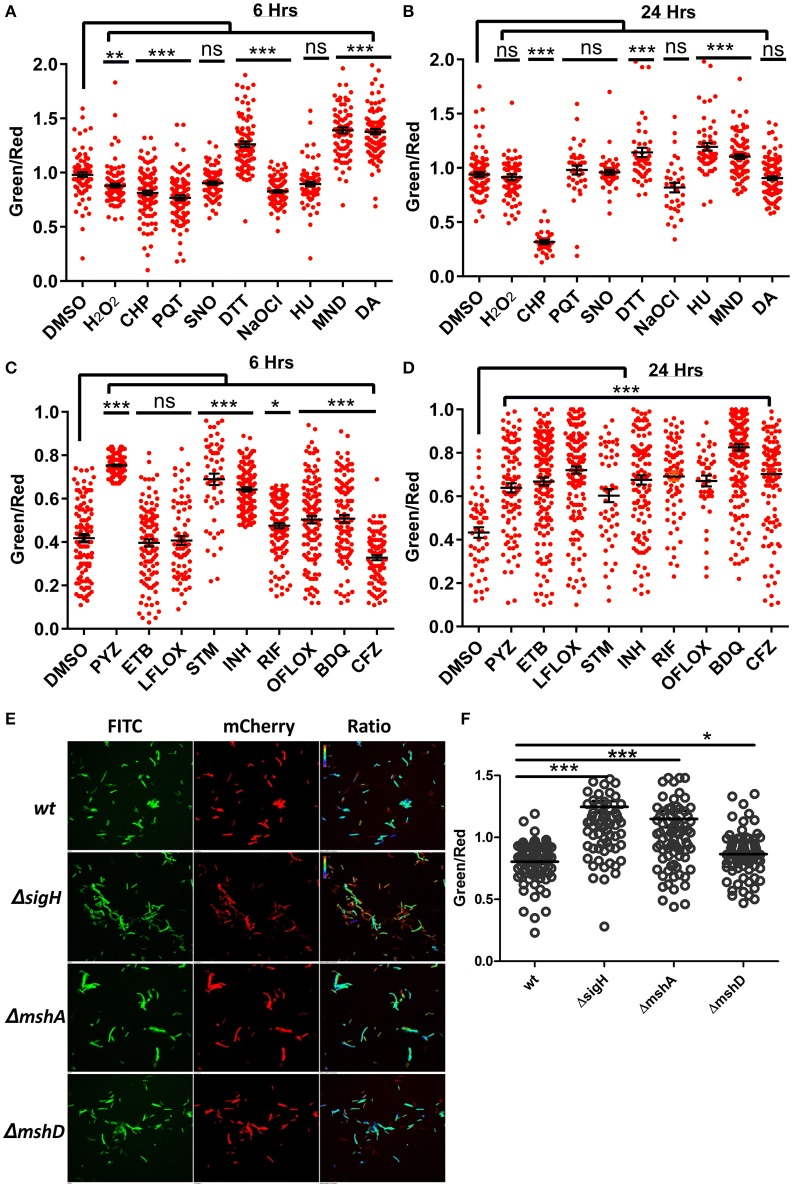
**Antibiotics and oxidoreductive reagents induced alterations in NADH:NAD+ ratios in mycobacteria. (A,B)** Upon treatment of reporter Mtb H37Rv cells with different oxidoreductants hydrogen peroxide (H_2_O_2_) (5 mM), cumene hydroperoxide (CHP) (5 mM), paraquat (PQT) (5 mM), Spermine NONOate (SNO) (1 mM), Dithiothreitol (DTT) (2 mM), sodium hypochlorite (NaOCl) (0.1%), hydroxyurea (HU) (10 mM), menadione (MND) (100 mM) and diamide (DA) (1 mM), for 6 and 24 h, confocal images were acquired and single cell ratios from confocal images were plotted in GraphPad Prism. **(C,D)** Scatter plots of single cell green:red ratio distribution in a population representing the changes in NADH:NAD^+^ ratios after treatment of H37Rv cells with antibiotics pyrazinamide (PYZ) (2 μg/ml), ethambutol (ETB) (5 μg/ml), levofloxacin (LFLOX) (0.5 μg/ml), streptomycin (STM) (2 μg/ml), isoniazid (INH) (0.2 μg/ml), rifampicin (RIF) (0.2 μg/ml), ofloxacin (OFLOX) (1 μg/ml), bedaquiline (BDQ) (0.35 μg/ml) and clofazimine (CFZ) (0.2 ug/ml), after 6 and 24 h post treatment. **(E)** Fluorimetric ratios (green/red) of the log phase cultures of the wild-type *Msmeg* reporter strain (Msm) compared to the mutant reporter strains *Msm*Δ*sigh*, Δ*mshA, and* Δ*mshD*. Data shown in **(A–D)** (mean ± SEM) is representative of three independent experiments performed in triplicate. Significance was determined by applying one one-way ANOVA by Dunnett's multiple comparison test, compared to control. ^*^p < 0.05, ^**^p < 0.01, ^***^p < 0.001 and ns, not significant.

After validating the capability of the Peredox in sensing the metabolic state of the fast-growing non-pathogenic mycobacteria (Msmeg), we transformed the IMT-100 expressing Peredox into the pathogenic Mtb H37Rv. The overexpression of Peredox in Mtb does not affect its growth, and thus this probe could be used for analyzing the metabolic state of Mtb *in vitro* and *in vivo*. In TB patients, Mtb primarily resides in alveolar macrophages, which produce bactericidal quantities of reactive oxygen (ROS), and reactive nitrogen intermediates (RNI) such as nitric oxide (NO), peroxynitrite, hydrogen peroxide (H_2_O_2_) and superoxide radical ${\text{O}}_{2}^{-}$, etc. Although these stresses are known to affect the metabolism of Mtb, their precise effect on NADH:NAD^+^ is not known. To analyze the effects of physiologically relevant stresses on the metabolic state of Mtb, we exposed Mtb cells overexpressing Peredox to the NO donor Spermine NONOate, hydrogen peroxide (H_2_O_2_), the H_2_O_2_ donor cumene hydroperoxide (CHP), the superoxide radical generators menadione (MND) and paraquat (PQT), hydroxyurea (HU), the hypochlorite anion (HOCl) donor sodium hypochlorite (NaOCl), the thiol reductant dithiothreitol (DTT) and the thiol oxidant diamide (DA) for 6 and 24 h, fixed the cells and then performed the CLSM analysis. Interestingly, we observed that after 6 h, NO had no effect on the ratio, whereas oxidants such as H_2_O_2_, CHP, MND, PQT and HOCl led to a decrease in the ratio, possibly due to oxidation of NADH in the cell (Figure 5A). After 24 h, the effect of H_2_O_2_, PQT and HOCl was abrogated, while in case of CHP, the NADH:NAD^+^ ratio further decreased. However, exposure to oxidants such as MND resulted in a higher NADH:NAD^+^ ratio (Figure 5B). We also checked the effect of thiol oxidative stress and thiol reductive stress on the levels of NADH:NAD^+^ of Mtb, using DA and DTT, respectively. Interestingly, we observed that both the agents led to increased levels of NADH:NAD^+^ (Figures 5A,B). In summary, Peredox effectively monitored changes in NADH levels in Mtb in response to diverse physiologically relevant redox stresses.

### Prolonged exposure to antimycobacterial drugs results in higher NADH:NAD^+^ levels

In the light of the fact that many of the antimycobacterial drugs are linked to energy metabolism of Mtb (Black et al., 2014; Koul et al., 2014; Maglica et al., 2015), measuring the levels of the NADH:NAD^+^ ratio becomes clinically important. Thus, we treated the Mtb Peredox with sub-lethal concentrations of antituberculosis agents for 6 and 24 h and calculated the NADH:NAD^+^ ratio though CLSM analysis. We observed that after 6 h of drug exposure, isoniazid (INH), rifampicin (RIF), pyrazinamide (PZA), streptomycin (STM), and ofloxacin (OFLOX) significantly increased the NADH:NAD^+^ levels (Figure 5C). We also observed for the first time using genetically encoded sensor that treatment with bedaquiline (BDQ) results in increased levels of NADH:NAD^+^. These changes in NADH levels perhaps result from the blockade of the ETC in producing ATP. However, Clofazimine treatment results in decreased levels of NADH:NAD^+^ levels during short-term exposure (Figure 5C). Interestingly, after 24 h of treatment with all tested antimycobacterials, a significant increase in the NADH:NAD^+^ level was observed (Figure 5D). The decrease in NAD^+^ concentration can be lethal to bacteria as NAD^+^ acts as a cofactor for many enzymes (Vilchèze et al., 2010), thus revealing the lethal effects of antibiotics that decrease oxidant nucleotide concentration. On the other hand, the increase in NADH concentration can be used for adapting to stressful conditions. Increased NADH can be used by the thioredoxin system for scavenging deleterious ROS produced within the cytoplasm due to antibiotics. However, it must be noted that increased NADH for a prolonged duration results in oxidation of NADH, producing a large amount of ROS, and damaging cellular components (Kohanski et al., 2007). These results link ROS production with the increase in the NADH:NAD^+^ ratio. To confirm this, we used a genetic knockout of *sigH (Msmeg*Δ*sigH*). SigH is the primary regulator of major anti-oxidant response systems in Mycobacteria; a sigH mutant displays significant downregulation of antioxidant systems (Raman et al., 2001) and is highly sensitive to oxidative stress and ROS. Analysis of the NADH:NAD^+^ in *Msmeg*Δ*sigH* revealed that this mutant has a significantly higher NADH:NAD^+^ ratio compared to the *WT* Msmeg (Figures 5E,F). We also analyzed the NADH:NAD^+^ in Msmeg mutants (*Msmeg*Δ*mshA and Msmeg*Δ*mshD*) in the mycothiol biosynthesis pathway. We observed that these mutants also exhibit significantly higher levels of NADH:NAD^+^ (Figures 5E,F).

### MTB residing inside macrophages exhibits higher NADH:NAD^+^ and metabolic heterogeneity

**Figure 6:**
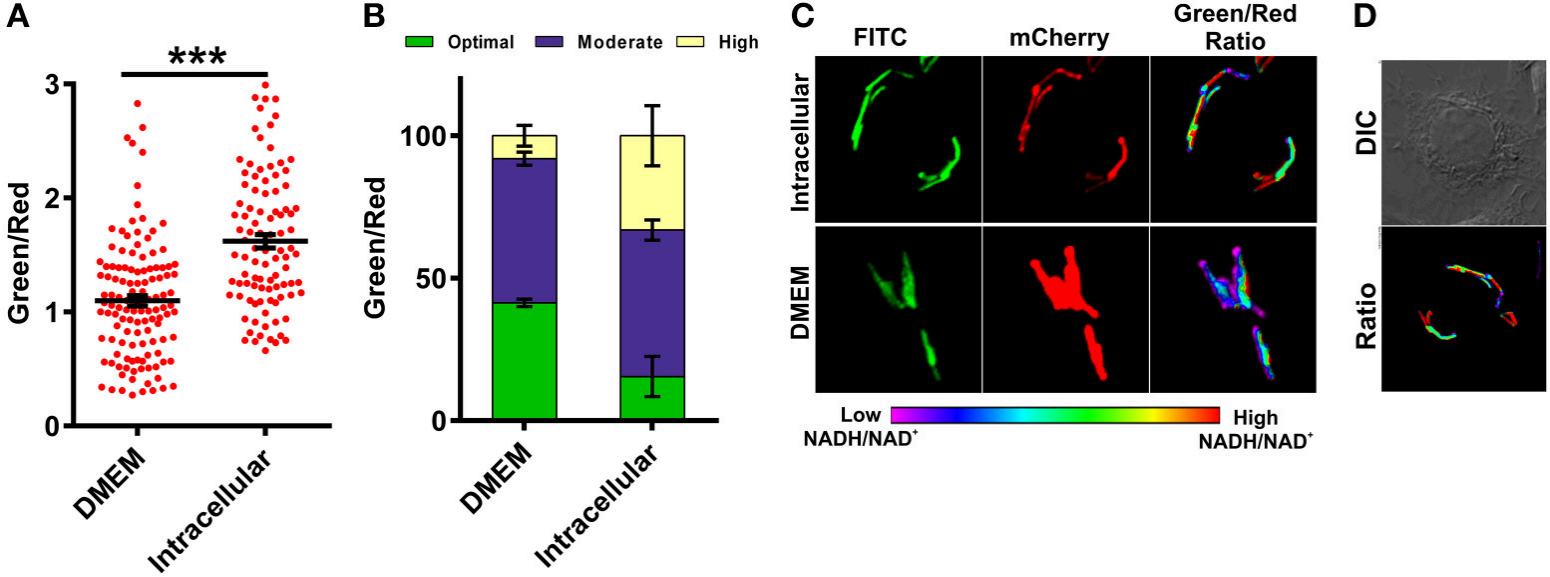
**Macrophages induce changes in mycobacterial NADH:NAD^**+**^ levels during infection**. Single cell ratios of the Mtb H37Rv reporter strain were determined either after incubating in DMEM or after infection in RAW 264.7 cells for 3 h. The data were acquired using confocal microscopy and green:red ratios were calculated and plotted as a scatter plot **(A)** or stacked bar plot **(B)** using GraphPad Prism® software. **(C)** Representative confocal images of bacteria in DMEM or inside RAW 264.7 macrophages. **(D)** DIC image representing infected cells with respective pseudo-colored ratiometric image. Error bars represent ± SEM and significance was determined using student's *t*-test, one tailed. ^***^p < 0.001.

The metabolic state of the Mtb during infection is poorly understood due to the absence of suitable tools by which to measure metabolism. To analyze the metabolic state of Mtb residing inside the macrophages, we infected RAW 264.7 macrophage cells with Mtb at a multiplicity of infection (MOI) of 1:10 and measured NADH:NAD^+^. We observed that Mtb residing inside macrophages had significantly higher NADH:NAD^+^ compared to extracellular Mtb cells growing in synthetic media (Figures 6A–D). Importantly, we also observed that Mtb residing in the macrophages exhibits considerable phenotypic heterogeneity compared to *in vitro* cultured Mtb cells (Figures 6A–D). The heterogeneity of Mtb residing inside macrophages indicates the distribution of Mtb in physiologically distinct niches. This heterogeneity could be one of the underlying reasons for the observed antitubercular drug tolerance in intracellular Mtb. For the purpose of comparison and analysis, we divided intracellular Mtb populations in the following groups (i) Mtb cells displaying optimal levels of NADH:NAD^+^ (comparable to NADH:NAD^+^ levels observed in *in vitro* cultures); (ii) Mtb cells displaying moderate NADH:NAD^+^ levels (cells having higher green/red); (iii) Mtb cells with reductive stress as depicted by having unusually high green/red and very high NADH:NAD^+^. It was observed that only a small fraction of intracellular Mtb cells possessed optimal NADH:NAD^+^. A larger number of intracellular Mtb cells possessed either moderate levels or very high levels of NADH:NAD^+^ (Figure 6). We believe that such high levels of NADH:NAD^+^ result from a slowdown of metabolism due to nutrient deprivation and oxidative stress exerted by macrophage cells.

### Interferon-γ activation of macrophage cells leads to increased NADH:NAD^+^ of intracellular MTB

**Figure 7:**
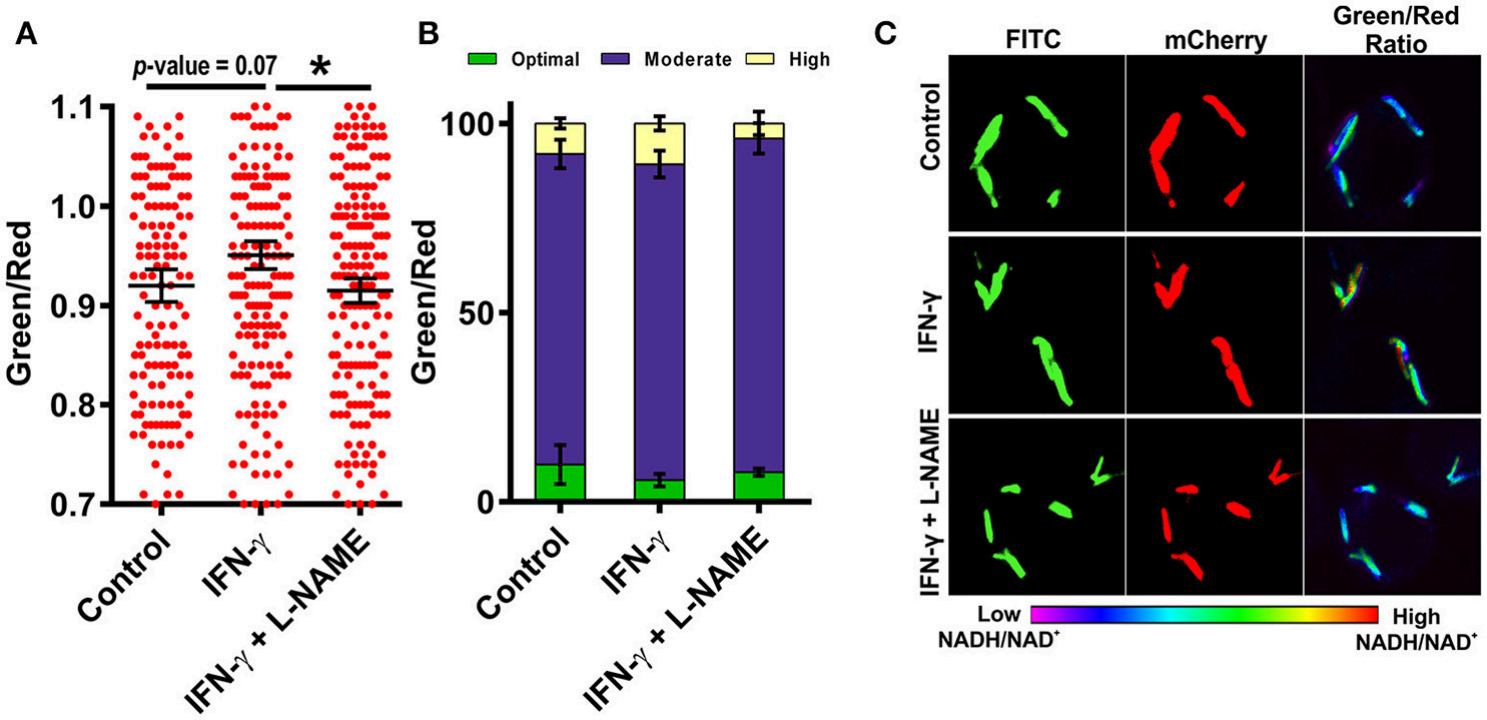
**Activation of macrophages induces changes in mycobacterial NADH:NAD^**+**^ levels during infection**. RAW 264.7 cells were infected with Mtb H37Rv Peredox reporter strain (MOI = 1:10) for 3 h after activation with IFN-γ or IFN-γ and L-NAME for 2 h. Samples were fixed with 4% PFA at 2 h post infection and analyzed by confocal microscopy. Single cell ratios (mean ± SEM) were calculated from confocal images using NIS elements analysis software and were plotted in a scatter plot **(A)** and stacked bar plot **(B)** showing the distribution and percentage heterogeneity of NADH:NAD^+^ ratios across the population. Inset in **(B)** shows the distribution of intracellular Mtb into groups exhibiting low, moderate and high levels of NADH:NAD^+^. **(C)** Representative confocal images of RAW 264.7 cells infected with Mtb H37Rv Peredox reporter strain after activation with IFN-γ alone or with L-NAME for 2 h. Data shown are representative of at least three experiments performed in triplicate. Error bars represent ± SEM, calculated using students one-tailed/two-tailed *t*-test. ^*^p < 0.05.

Resting macrophages are not able to inhibit the growth of intracellular Mtb but activated macrophages can restrict the growth of intracellular Mtb through the generation of ROS, RNS and by reverting the arrest of phagosome maturation (Schaible et al., 1998). To analyze the effect of immunological stimulation of macrophages on the metabolism of Mtb, we stimulated murine macrophages with the cytokine interferon gamma (IFN-γ) and then infected the cells with Mtb at an MOI of 1:10 and analyzed the NADH:NAD^+^ levels of the intracellular Mtb using CSLM. Interestingly, Mtb cells residing in the IFN-γ treated macrophages had higher NADH:NAD^+^ levels compared to the Mtb cells residing in the naive macrophages (Figures 7A–C). This increase was consistently observed in multiple experiments. We also observed that the population with the optimal levels of NADH:NAD^+^ shrank and the population displaying moderate and higher NADH:NAD^+^ increased, suggesting that treatment with IFN-γ influences the metabolic state of the intracellular Mtb. Because inducible nitric oxide synthase (iNOS) generated NO plays a critical role in regulation of Mtb growth in infected macrophages, we next analyzed the effects of macrophage generated NO on the metabolic state of intracellular Mtb. We employed L-NG-Nitroarginine methyl ester (L-NAME) which is a selective inhibitor of iNOS (Griffith and Kilbourn, 1996). Interestingly, the NADH:NAD^+^ levels of the Mtb cells residing in L-NAME treated IFN-γ stimulated macrophages were similar to the Mtb cells residing in the un-stimulated macrophages (Figures 7A–C) suggesting a major role of NO in the modulation of NADH:NAD^+^ levels in intracellular Mtb. These data indicated that the NADH:NAD^+^ levels of intracellular Mtb are tightly regulated in response to changes in the microenvironment.

### Effect of antimycobacterial drugs on the NADH:NAD^+^ of intracellular MTB

**Figure 8:**
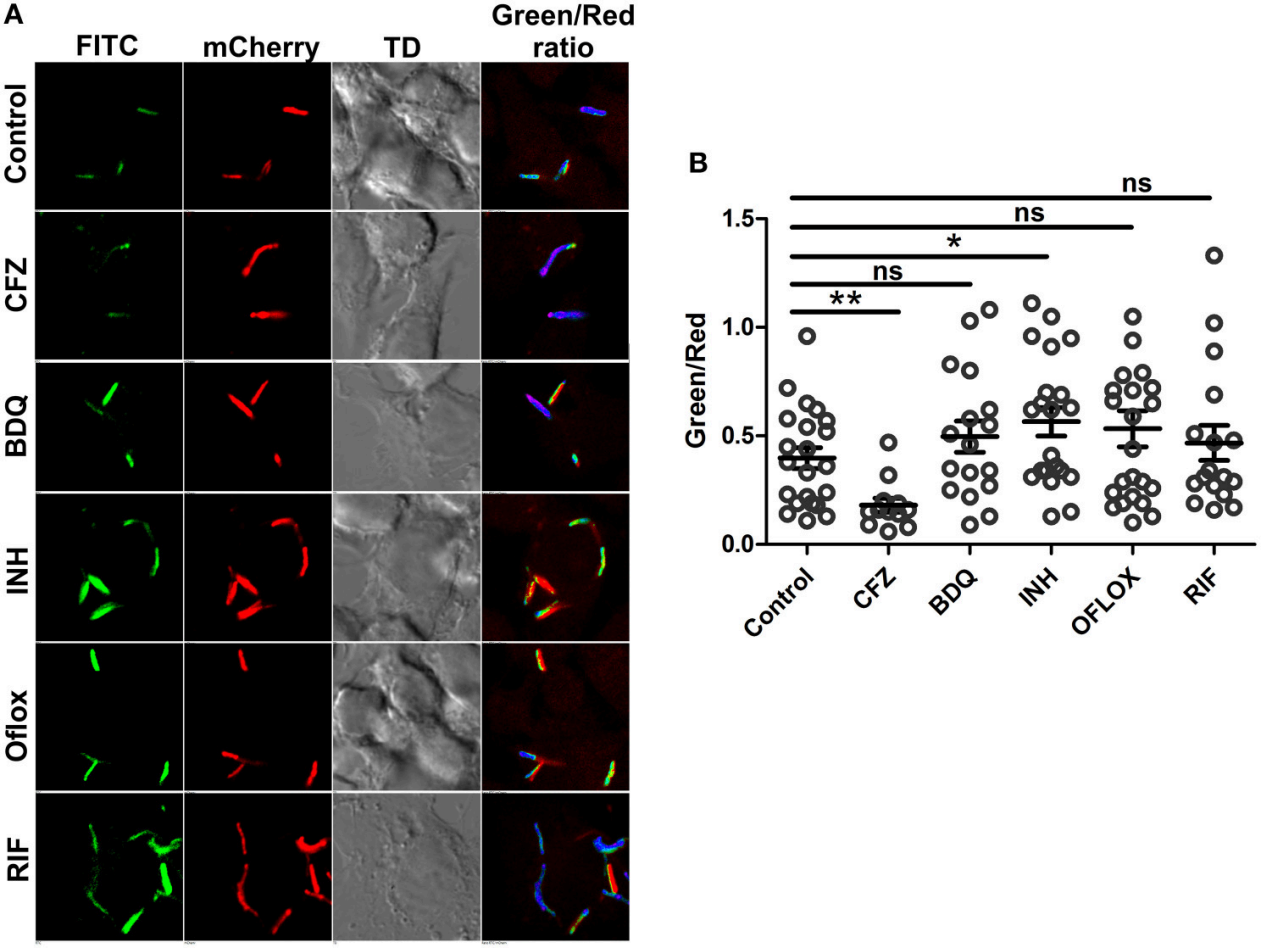
**Intracellular Mtb modulate change in NADH:NAD^**+**^ ratios in response to antibiotics**. The green/red ratios corresponding to NADH:NAD^+^ ratios of Mtb H37Rv reporter strain were determined either after 12 h of exposure to drugs post infection in RAW 264.7 cells (MOI = 10). Data were acquired using confocal microscopy. **(A)** Representative confocal images of intracellular with their respective pseudo-colored ratiometric image **(B)** Green:red ratios were calculated from confocal images using NIS Analysis software from Nikon and plotted as a scatter plot using GraphPad Prism® software. Error bars represent ± SEM and significance was determined using student's *t*-test, one tailed. ^*^p < 0.05, ^**^p < 0.01.

Earlier study by Bhasker et al has suggested that antimycobacterial drugs induce oxidative stress in intracellular Mtb (Bhaskar et al., 2014), while using Mtb peredox reporter strains, we have established that exposure to antimycobacterial agents induce stress wherein the levels of NADH are higher. The effect of antimycobacterial drugs on the NADH:NAD^+^ redox homeostasis of intracellular Mtb has not been studied, hence, we infected RAW 264.7 macrophage cells with Mtb Peredox and measured the NADH:NAD^+^. After 12 h post infection, we observed that INH led to significantly higher levels of NADH:NAD^+^ in infecting Mtb compared with the untreated intracellular Mtb (Figures 8A,B). Other antimycobacterial such as BDQ, OFLOX and RIF also consistently increased the levels of NADH:NAD^+^, however, this increase was not statistically significant (Figures 8A,B). It could be presumed that the antibiotic exposure inhibits the metabolism of intracellular Mtb that results in accumulated levels of NADH. The accumulated levels could later generate ROS that could result in lethality in Mtb. These studies are also consistent with an earlier study that has suggested increased levels of NADH:NAD^+^ in the intracellular Mtb upon exposure to BDQ (Koul et al., 2014). On the other hand, exposure of intracellular Mtb to the Clofazimine repeatedly resulted in significantly decreased levels of NADH:NAD^+^ (Figures 8A,B). These data are particularly interesting in the light of capability of clofazimine to catalyze NADH-dependent redox cycling for ROS production (Yano et al., 2011).

## Discussion

NADH:NAD^+^ is considered the metabolic readout of bacterial cells; however, current methods for its detection do not provide spatiotemporal resolution and thus are unsuitable for monitoring metabolism at the single-cell level. Due to these reasons, measurement of NADH:NAD^+^ levels in pathogens is not amenable during complex and dynamic interactions with macrophage cells. In this study, we have exploited a simple, non-invasive, genetically encoded sensor derived from the NADH sensor Rex to examine the mycobacterial NADH:NAD^+^ redox state in real time. To the best of our knowledge, the data presented here are first to quantify the NADH:NAD^+^ redox state of fast and slow-growing Mycobacterium at single cell levels under various physiologically relevant stresses such as oxidative and nitrosative stress and stress generated through the use of antimycobacterial drugs. This study has further demonstrated that Mtb residing in macrophages display a higher level of NADH:NAD^+^ and increased heterogeneity compared to bacteria growing *in vitro*. We have also demonstrated for the first time that this metabolic state of intracellular Mtb is perturbed in response to immunological modulation of the macrophages.

One of the major accomplishments of this study is the exploitation of the Peredox probe to trace the levels of NADH:NAD^+^ in Mtb cells during infection. Interestingly, significantly higher levels of NADH:NAD^+^ were observed in the intracellular bacteria compared to the Mtb cells growing in synthetic culture media. A plausible explanation for these higher levels of NADH:NAD^+^ is that synthetic culture media is designed to facilitate maximal growth via the upregulation of metabolic pathways that generate a higher pool of NAD^+^ for efficient harvesting of electrons. Inside phagosomes, bacteria are faced with nutrient deprivation and metabolic slowdown that could lead to accumulated NADH levels. These results are consistent with the increased duplication time of intracellular bacteria (Rohde et al., 2012). A similar increase in intracellular NADH:NAD^+^ level was also observed in Mtb recovered from lungs of infected mice or from macrophages (Boshoff et al., 2008). These findings point to a metabolic reprogramming of Mtb upon the infection of macrophages. A similar metabolic reprogramming was also suggested by transcriptome profiling of Mtb residing in macrophages (Schnappinger et al., 2003). Studies have suggested that higher levels of NADH:NAD^+^ could lead to an overexpression of the virulence factor serine/threonine kinase PknG, which plays an important role in the intracellular survival of Mtb (Wolff et al., 2015). Moreover, it was recently demonstrated that thiol reductive stress induces Mtb biofilm formation (Trivedi et al., 2016). There is a significant heterogeneity in the metabolic state of bacterial cells residing in different spatial location inside a biofilm or during infection. However, in the absence of tools like peredox this metabolic heterogeneity was in accessible; further utilization of this novel tool will facilitate such studies. We believe that with development of this tool, now the NADH;NAD^+^ in different cells of Mtb biofilms could be examined. Another important observation from this study was a higher metabolic heterogeneity in the intracellular bacteria compared to the *in vitro* growing bacteria. This observation is consistent with earlier findings reporting heterogeneity in the mycothiol redox potential of intracellular Mtb and its correlation with the resistance toward antimycobacterials (Bhaskar et al., 2014). This metabolic heterogeneity could also be one of the underlying reasons for the drug tolerance observed in intracellular Mtb, but such a possibility remains to be further analyzed. In line with this hypothesis, earlier studies have suggested that higher NADH levels could lead to resistance against INH and ETH (Vilchèze et al., 2005). Another important finding from this study was that Mtb cells residing in the IFN-γ activated macrophage cells experience higher metabolic stress compared to the ones residing in the naïve macrophages. These findings could be due to the capability of Mtb to inhibit the fusion of phagosomes with the lysosomes in naïve macrophages, however upon stimulation with IFN-γ, this blockage could be averted. Thus, Mtb residing inside the IFN-γ activated macrophages faces higher metabolic stress. These findings are consistent with earlier studies wherein; virulence factors such as phosphatase ptpA (Wong et al., 2011) and serine/threonine kinase pknG (Walburger et al., 2004) could alter recruitment of vacuolar-H^+^- ATPase to inhibit acidification of phagolysosomes and thus survival of mycobacteria in macrophages. Importantly, recent studies demonstrated that Mtb cells residing in phagosomes are exposed to a lower pH and a higher concentration of Cl^−^ ions (Tan et al., 2013). Another explanation of these findings could be that upon stimulation with IFN-γ, macrophages produce higher amounts of NO, which could inhibit the respiration of intracellular Mtb cells. This inhibition of respiration may result in higher levels of NADH:NAD^+^.

We have also utilized the Mtb Peredox reporter strain to understand the effect of physiologically relevant stresses on Mtb cells in *in vitro* cultures. These studies are significant in that they demonstrate the capability of the Peredox probe to define the effect of a specific stress on NADH:NAD^+^ in Mtb cells. Interestingly, we observed that short exposure to oxidative stress by H_2_O_2_, PQT and HOCl leads to decreased NADH:NAD^+^ levels, but after extended exposure, Mtb cells are able to adapt to these stresses and maintain NADH:NAD^+^ levels. The capability of Mtb cells to maintain NADH levels in the presence of nitrosative stress was surprising because NO is known to inhibit cellular respiration through inhibition of the ETC (Voskuil et al., 2003). The metabolic pathways that ensure maintenance of NADH:NAD^+^ levels in the presence of NO remain to be studied and were beyond the scope of the present study. We have also evaluated the effect of antitubercular agents on Mtb metabolism and observed that exposure to antitubercular drugs such as results in the accumulation of NADH, likely due to the inhibition of metabolism. These data were consistent with previously reported transcriptome changes suggesting metabolic shutdown upon exposure to antitubercular agents (Boshoff et al., 2004). Furthermore, we have analyzed the effect of antimycobacterial agents on the NADH:NAD^+^ levels of Mtb residing in the macrophages. We have observed that antimycobacterial agents such as INH, BDQ, OFLOX and RIF induce a metabolic stress that results in the accumulation of NADH. We believe that increase in the levels of NADH:NAD^+^ is a metabolic response to the antimycobacterials. Interestingly, a number of studies have suggested that increased levels of NADH:NAD^+^ could assist Mtb cells to exert resistance against the antimycobacterial drugs (Rozwarski et al., 1998; Vilchèze et al., 2005; Rodionova et al., 2014). Importantly, Clofazimine was found to decrease the NADH:NAD+ levels. However, further experiments are required to establish if sub-lethal levels of the Clofazimine could assist killing of Mtb cells by drugs such as INH and ETA which becomes less effective due to increased NADH:NAD^+^. Notably, we have also established a link between levels of the mycobacterial redox buffer mycothiol and NADH:NAD^+^ using mutant strains incapable of mycothiol biosynthesis. The elevated NADH:NAD^+^ observed in *Msmeg*Δ*mshA* could potentially explain the observed isoniazid and ethionamide resistance in this mutant strain (Xu et al., 2011). However, the higher levels of NADH:NAD^+^ in *Msmeg*Δ*mshA* do not agree with the lower level of NADH observed in MtbΔ*mshA* (Vilchèze et al., 2008). This deviation could be because different species were evaluated or due to different methods of measurement between the two studies. Given the role of NADH:NAD^+^ in resistance to isoniazid and ethionamide, Peredox could be used in high content screening for small-molecule inhibitors that decrease NADH:NAD^+^ levels. Identification of such inhibitors will facilitate the creation of novel antitubercular agents that could synergize with isoniazid or ethionamide. We have also utilized the Peredox probe to measure the effect of inhibitors of the ETC on the NADH levels. We consistently observed that inhibition of ETC results in increased NADH:NAD^+^ levels. These results suggest that the ETC is the primary consumer of NADH, although other ways to consume NADH (Trivedi et al., 2012) or degrade NADH (Wolff et al., 2015) exist in mycobacteria.

## Author contributions

SB and AK designed research. SB and II performed research, acquired and analyzed data. SB and AK interpreted data and wrote the manuscript.

### Conflict of interest statement

The authors declare that the research was conducted in the absence of any commercial or financial relationships that could be construed as a potential conflict of interest.
