# Supplementary figures and images for: Imaging the NADH:NAD+ Homeostasis for Understanding the Metabolic Response of Mycobacterium to Physiologically Relevant Stresses
Source: Front Cell Infect Microbiol. 2016 Nov 8;6:145. doi: 10.3389/fcimb.2016.00145 (PMC5099167; doi:10.3389/fcimb.2016.00145)

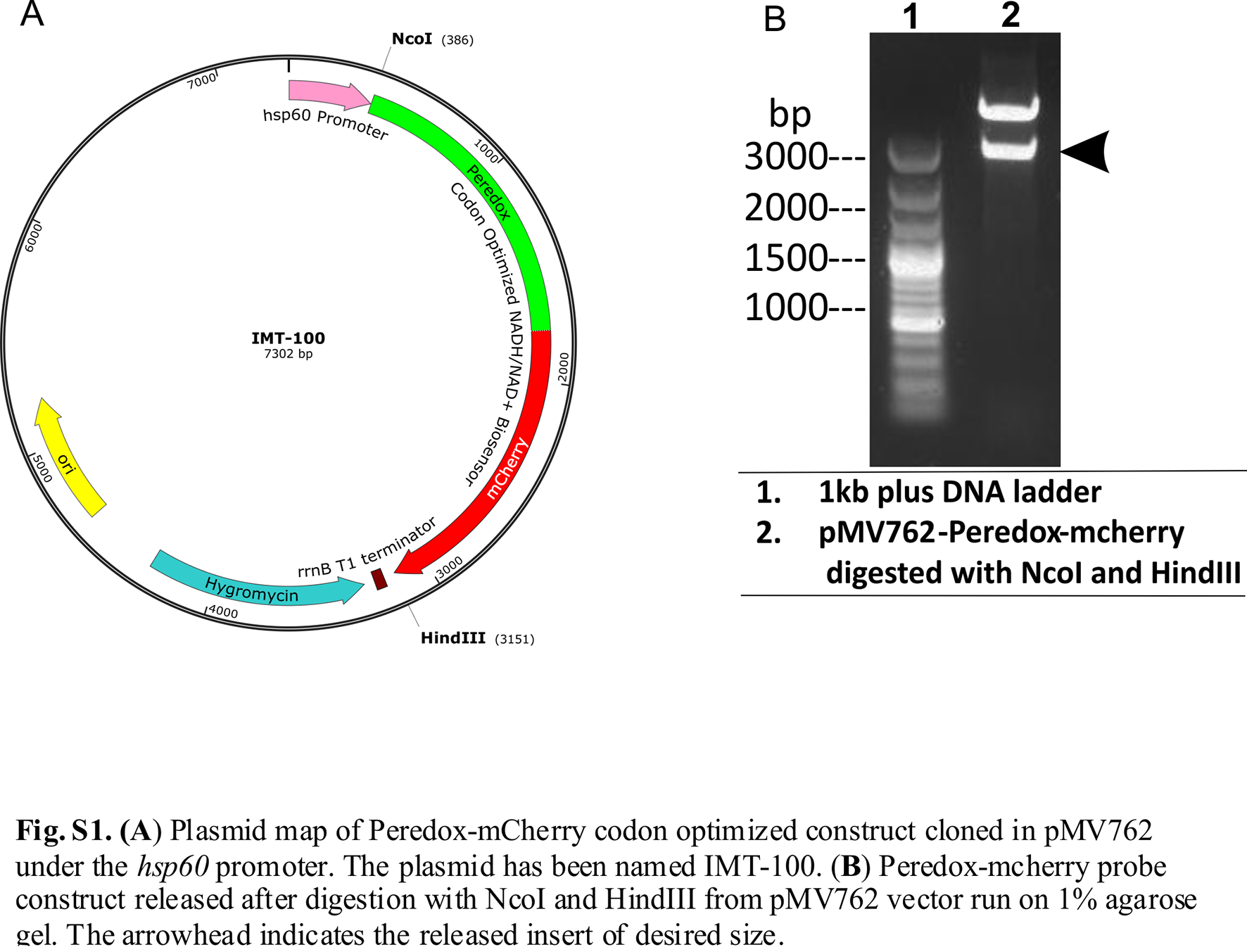

Supplement: Supplementary file 1 [file Image1.TIF]

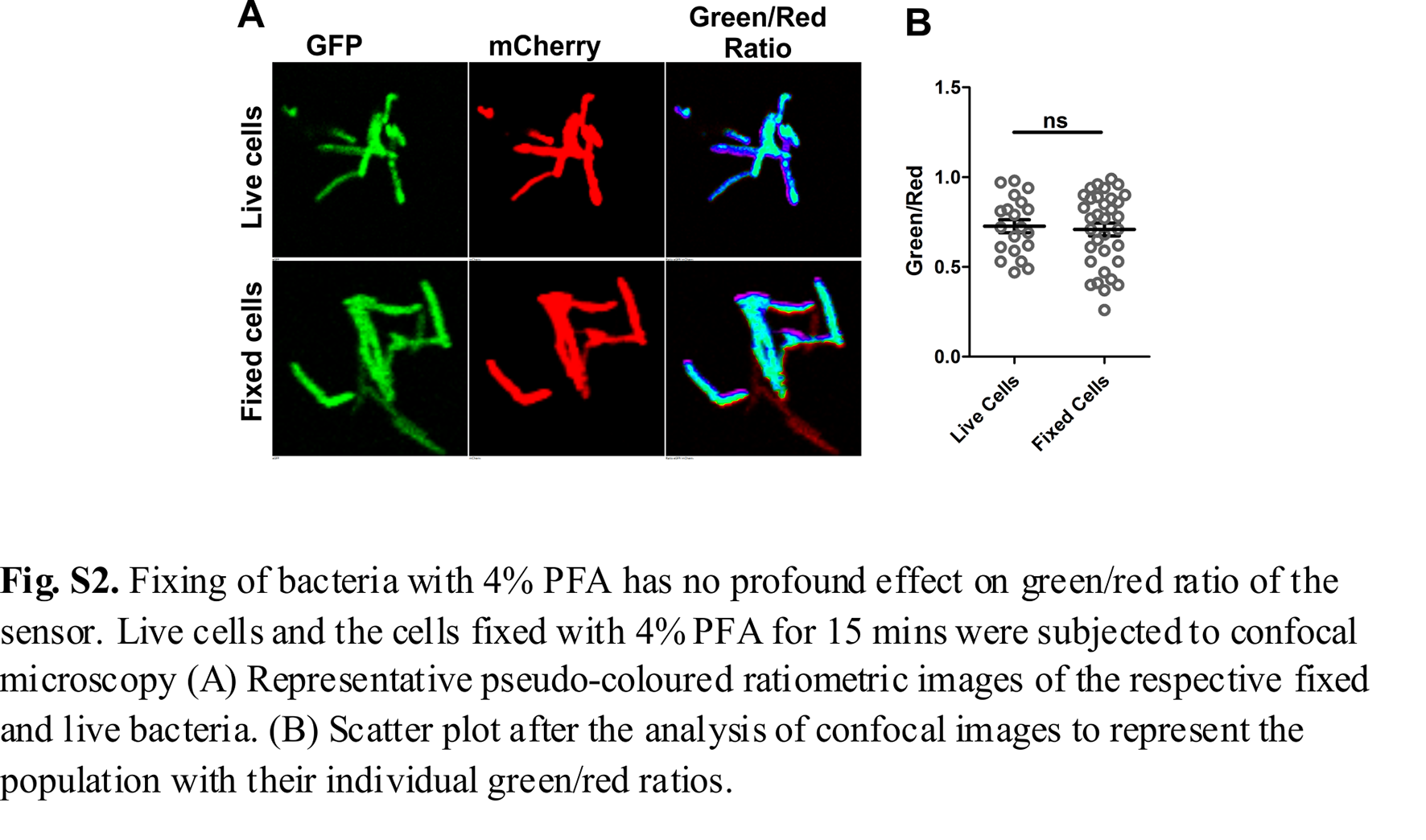

Supplement: Supplementary file 2 [file Image2.TIF]
